# Researches on Inflammation and Suppuration

**Published:** 1871-12

**Authors:** 


Researches on Inflammation and Suppuration.—At a meeting of the Royal Irish Academy, held on May 8th, Dr. J. M. Purser read the second part of his report on the above subject. It is reported by the *British Medical Journal*, which gives a very full account of the paper. Herr Cohnheim believes that the two following propositions are established as the result of his investigations: 1. In an inflamed part, the white corpuscles of the blood pass through the walls of the vessels in great numbers, and, having become free in the tissue, constitute the cells of pus. 2. The cells of the inflamed part itself have no share in the formation of pus; they persist for a time unchanged among the emigrated blood-corpuscles, and, if the inflammation last long enough, or attain a great intensity, they undergo a series of changes of a purely regressive or degenerative nature, ending in their death or destruction. Dr. Purser, in the first part of his report, read twelve months since, stated that his own observations fully bore out Professor Cohnheim's views as enunciated in the first of the above quoted propositions. So far back as the year 1846, Dr. Augustus Waller had described the passage of the leucocytes of the blood through the walls of the vessels. With regard to the second proposition of the German physiologist, however, Dr. Purser found that the experiments conducted by himself gave negative results, and in them he was borne out by the opinions of Virchow and of Goodsir. Having described Professor Cohnheim's mode of procedure in experimenting on the corneæ of frogs, Dr. Purser proceeded to give in detail the results which he had himself obtained. His observations were also made on the corneæ and tongues of frogs. Inflammation was excited either by cauterization with nitrate of silver, or by the insertion of a seton. In some instances, the occurrence of a spontaneous ulcerative keratitis obviated the necessity of causing irritation. Phenomena, essentially the same in kind, but varying much in degree and as to the time of their development, showed themselves in every case. On no occasion did the connective-tissue cells remain unaltered among the pus-corpuscles. The first well-marked change observed in the former consisted in a tendency to become elongated, and, in doing so, to lose their equally stellate shape. Their nuclei underwent a similar modification of form, and the protoplasm assumed a more decidedly granular appearance than in health. In the next stage of the inflammatory process, the cells have completely lost their primitive form, and have become perfect spindle-shaped bodies, while the number of nuclei increased, and amounted sometimes to four or more in a single cell. The third change consisted in the division of the spindle-shaped corpuscles. These first assumed an hour-glass appearance, and finally divided across in one or more than one place. Sometimes the spindles did not divide, but formed movable, multi-nucleated masses, like those described by Stricker. Dr. Purser believed, too, that the researches of this physiologist on inflammation confirm his own observations.—*London Microscopical Journal.*

